# Electronic and Magnetic Structures of New Interstitial Boron Sub-Oxides B_12_O_2_:X (X = B, C, N, O)

**DOI:** 10.3390/molecules26010123

**Published:** 2020-12-29

**Authors:** Samir F. Matar, Jean Etourneau

**Affiliations:** 1 Lebanese German University, Sahel-Alma, Jounieh 1200, Lebanon; 2 University of Bordeaux, ICMCB-CNRS, 33600 Pessac, France; jean.etourneau@icmcb.cnrs.fr

**Keywords:** *p*-magnetism, boron sub-oxide, interstitial atoms, DFT, DOS, ELF, charge density plots

## Abstract

The boron-rich boron sub-oxide rhombohedral B_6_O considered in B_12_O_2_ full formulation has a large O-O spacing of ~3 Å and a central vacant position that can receive interstitial atoms X, forming a central O-X-O alignment in the dodecaboron cage as observed in well-known triatomic B_12_ compounds as B_12_{C-C-C}, B_12_{N-B-N}, etc. Plane wave density functional theory (DFT) based calculations of unrestricted geometry relaxation of B_12_{O-X-O}, X = B, C, N, and O let one identify new ternary sub-oxides, all found cohesive while showing different d(X-O) distances ranging from d(B-O) = 1.95 Å down to d(O-O) = 1.73 Å with intermediate d(C-O) = 1.88 Å. The different magnitudes were assigned to the chemical affinities of X-inserts versus host oxygen with the increasing development of X-O bonding along the series with larger cohesive B_12_{O-O-O}. From the atom projected charge density, B presents none, while significant magnitudes are shown on C and N, the latter developing bonding with terminal oxygen atoms especially N. The presence of unpaired valence electrons leaves nonbonding charge density on X = C, N interstitial compounds, which, besides the relative isolation of the central C and N lead to the onset of magnetic moments: M(C) = 1.9 μ_B_, and M(N) = 1 μ_B_ in a ferromagnetic ground state. Atom-resolved assessments are provided with the magnetic charge density and electron localization function electron localization function (ELF) projections on one hand and the site and spin projected density of states and the chemical bonding based on the overlap integral S_ij_ within the COOP criterion, on the other hand.

## 1. Introduction

**Figure 1:**
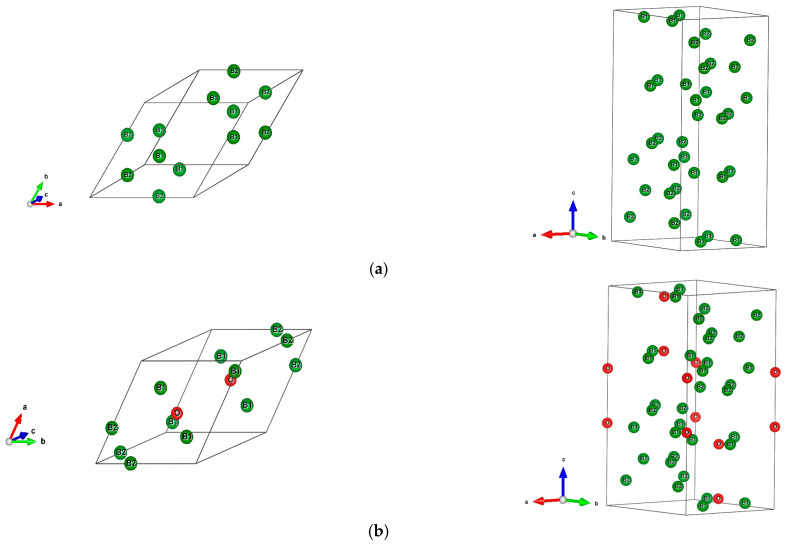

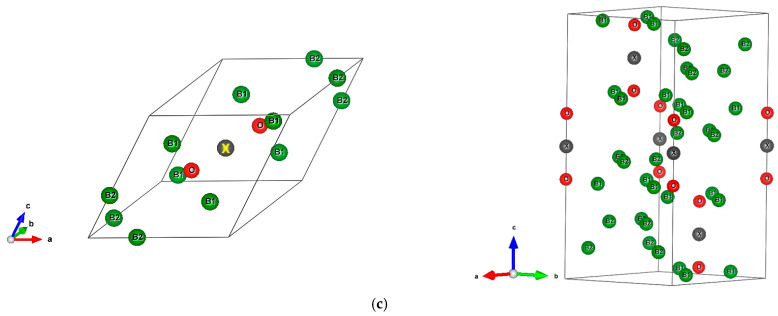
(color). Representation of the B_12_-based structures in rhombohedral (left) and hexagonal (right) settings, with 1 and 3 formula units (FU) respectively. (**a**) α-B_12_, (**b**) B_12_O_2_, (**c**) Proposed B_12_O_2_:X, with X representing a generic interstitial at the central void (X = B, C, N, and O).

The sesquioxide B_2_O_3_ is the best known boron oxide and its hydration leads to the well-known boric acid B(OH)_3_, which has many uses in the medical sector as an antibacterial and in chemistry as a pH buffer. However, more relevant to solid state chemistry is its use in combination with boron to build the B_2_O_3_-B phase diagram [1]. Boron rich compounds were identified as B_6_O [2] characterized by a structure resembling the simplest form of boron, α-B_12_ (space group *R*$\bar{3}$*m*, N°166) [3]. A small amount of oxygen sub-stoichiometry was identified by Olofsson and Lundström [4] who claimed that larger oxygen content can be attained at pressures above ambient conditions. For the sake of complete review, the B_2_O composition with trigonal structure was proposed as an unsymmetrical analog of carbon by Endo et al. [5], but its structure as a carbon derived one was deemed as unstable by density functional theory (DFT) total energy calculations [6]. The quantum theoretical DFT framework [7,8] is also used in the present investigative work. The B_12_ structure depicted in Figure 1a in both rhombohedral (1 formula unit FU) and hexagonal (3 FU) settings, shows a remarkable vacant space surrounded by 6 B1, that can be occupied by one, two, and/or three interstitial atoms leading to a large family of compounds (cf. [9] for a review on boron with enumerated families). B_6_O has the same space group as α-B_12_ and it can be expressed as B_12_O_2_ in fully stoichiometric formulation that we consider here as the host of foreign insertion elements (vide infra). In the structure shown in Figure 1b, oxygen atoms occupy the two-fold Wyckoff positions (2*c*) in the vicinity of B1 while the B2 atoms are pushed farther along the diagonal towards the rhombohedron corners as with respect to their positions in B_12_. In the hexagonal setting, oxygen atoms align along c*_hex_*. Concomitantly, the rhombohedral angle α_rh._ = 63.2° is enlarged with respect to its magnitude in B_12_ where α_rh._ = 58°, i.e., there is an opening of the rhombohedron to receive the interstitials. A relevant feature resulting from the 3B1-O bonding is the large spacing between the two oxygen atoms, amounting to ~3 Å, highlighting their isolation from each other on one hand and offering the central void at the body center defined by the Wyckoff position 1*b* (½,½,½) on the other hand. In the context of the present investigation, the central position is made to receive interstitials of light elements called X, leading to express B_6_O as B_12_O_2_:X depicted in Figure 1c in both rhombohedral and hexagonal settings. Note that this formulation highlighting central triatomic linear alignments is identified in other compounds as the recently investigated B_13_N_2_ expressed as B_12_N_2_:B [10,11] as well as B_12_C_2_:X (X = B, C) [12,13]. Consequently, the present paper has the purpose of presenting investigation results of original compounds B_12_O_2_:X considering herein a series of neighboring p-interstitial light elements: B, C, N, and O studied within the DFT. The original compounds belonging to the large family of α-B_12_ based chemical compounds will be shown to be cohesive and possessing particular electronic properties as well as magnetic ones for some of them. They are proposed to further broaden the scope of boron research in chemistry and physics. For the sake of clear and simple presentation, the results are presented for 1 FU within the rhombohedral setting.

## 2. Computational Framework

The search for the ground state structure and energy was carried out within DFT based calculations using plane-wave code Vienna Ab initio Simulation Package VASP [14,15] with the projector augmented wave (PAW) method [15,16] for the atomic potentials with all valence states especially in regard of the light elements B, C, N, and O. The exchange-correlation XC effects within DFT were considered with the generalized gradient approximation (GGA) [17]. This XC scheme was preferred to the homogeneous gas-based local density approximation (LDA), one which led in preliminary calculations to underestimated structure parameters, indeed, LDA is known to be over-binding [18]. The conjugate-gradient algorithm [19] was used in this computational scheme to relax the atoms onto the ground state. The tetrahedron method with Blöchl et al. corrections [20] and Methfessel–Paxton [21] scheme was applied for both geometry relaxation and total energy calculations. Brillouin-zone (BZ) integrals were approximated using a special **k**-point sampling of Monkhorst and Pack [22]. The optimization of the structural parameters was performed until the forces on the atoms were less than 0.02 eV/Å and all stress components were below 0.003 eV/Å^3^. The calculations converged at an energy cut-off of 500 eV for the plane-wave basis set concerning the **k**-point integration with a starting mesh of 6 × 6 × 6 up to 12 × 12 × 12 for best convergence and relaxation to zero strains. Calculations are firstly carried out considering total spins configuration, pertaining to non-spin polarized (NSP) configurations. In a further step, due to the paramagnetic character observed from the valence electron count (VEC, see below), spin-polarized SP calculations were carried out.

**Figure 2:**
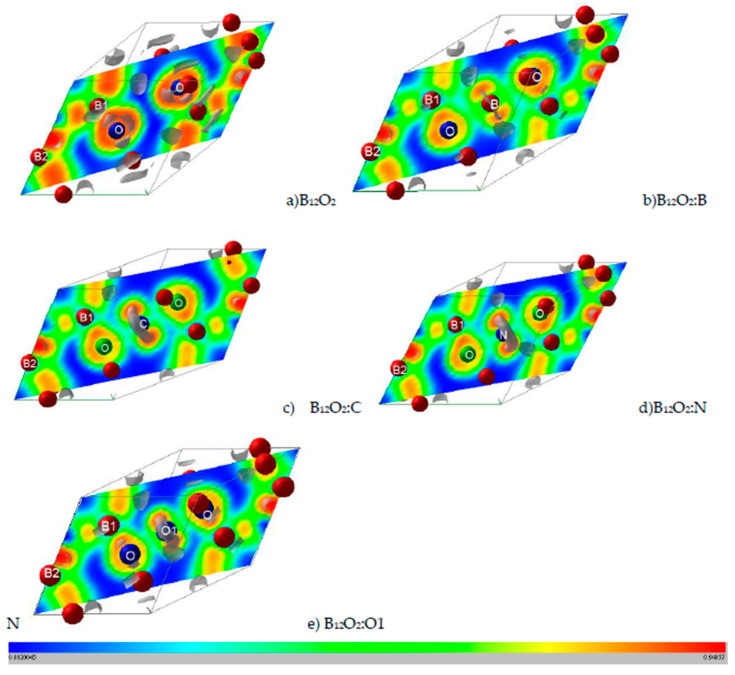
(color). ELF slice planes along diagonal plane. (**a**) B_12_O_2_, (**b**) B_12_O_2_:B, (**c**) B_12_O_2_:C, (**d**) B_12_O_2_:N, and (**e**) B_12_O_2_:O or B_12_O_3_. The ruler shows the color code with blue, green, and red corresponding respectively to 0 (no electron localization), ½ (free electron like), and 1 (full electron localization). Grey volumes depict 3D ELF, especially for showing the non-bonded electrons around central inserted atoms: C, N, and O.

Properties related to electron localization were obtained from real-space projections of the electron localization function (ELF) according to Becke and Edgecomb [23] and Savin et al. [24] as based on the kinetic energy in which the Pauli Exclusion Principle is included. ELF is a normalized function, i.e., 0 < ELF < 1, ranging from no localization for 0 (blue zones) and full localization for ELF = 1 (red zones) and free-electron gas behavior corresponds to ELF = ½ (green zones), cf. Figure 2.

In the post-treatment process of the ground state electronic structures, the total charge densities “CHGCAR”, as well as the magnetic charge density “CHGCAR_magn” are illustrated. The latter is computed if spin polarized SP calculations identify a magnetic solution, versus nonmagnetic NSP configuration.

From the geometry of the ground state structures NSP and SP, the detailed electronic site and spin projected density of states (PDOS) were obtained within DFT using full potential augmented spherical wave (ASW) method [25] and the GGA for the XC effects [17]. Also, the properties of chemical bonding are qualitatively assessed within ASW based on overlap matrix (S_ij_) with the crystal orbital overlap population (COOP) criterion [26]. In short hand notation, the COOP’s are the S_ij_—weighted density of states (DOS). Positive, negative and zero COOP magnitudes (cf. Section 4) correspond to bonding, anti-bonding and non-bonding interactions.

In the minimal ASW basis set, the outermost shells were chosen to represent the valence states and the matrix elements. They were constructed using partial waves up to *l_max_* = 1 for B, O, and X p-elements interstitials. In the most electronegative element, oxygen’s low energy lying 2s states were omitted from the valence basis set in the DOS projection for the sake of clarity. Self-consistency was achieved when charge transfers and energy changes between two successive cycles were: ΔQ < 10^−8^ and ΔE < 10^−6^ eV, respectively. The Brillouin-zone integrations were performed using the linear tetrahedron method within the irreducible rhombohedral wedge following Blöchl et al. scheme [20].

## 3. Results from Energy Calculations

### 3.1. Trends of Cohesive Energies

Firstly, we examined the B_6_O based compounds for their respective cohesive energies obtained considering total spin configuration within unconstrained, parameter-free, successive self-consistent sets of calculations at an increasing number of **k**-points. The structural results are shown in Table 1 and they will be discussed in the next section, focusing here on the cohesive energies deducted from subtracting the constituents’ atomic energies, averaged per atom to enable comparison between the different stoichiometries. The atomic constituents’ atomic energies in eV are as follows: E(B) = −5.56; E (C) = −6.48; E(N) = −5.2; and E(O) = −3.14.

Pure *rh.*α-B_12_ was identified with E_coh_./at. = −1.16 eV. Comparatively, B_12_O_2_ (B_6_O) was found with a larger cohesive energy: E_coh_./at. = −1.97 eV, explained by the selective bonding of B1 with the two oxygen atoms, the B1-O bond introducing an iono-covalent character.

Upon insertion with B, C, and N the cohesive energies: E_coh_./at.(B_12_O_2_:B) = −1.280 eV; E_coh_./at.(B_12_O_2_:C) = −1.266 eV and E_coh_./at.(B_12_O_2_:N) = −1.234 eV are all intermediate between α-B_12_ and B_12_O_2_. While they all remain within a close range of magnitudes, their decrease from B to C, and N is likely due to the decreasing difference of electronegativity χ between O and X: χ_O_ = 3.44, χ_B_ = 2.04, χ_C_ = 2.55, and χ_N_ = 3.04. This progressive decrease of the ionic character Δχ(X-O) is concomitant with a slight decrease of the cohesive energy. However, the relatively large decrease of the cohesive energy from pristine B_12_O_2_ to B_12_O_2_:X is assessed through the competitive B1-O versus X-O bonding along with the decrease of d(X-O) within the series as shown in Table 1.

An exception is nevertheless observed upon inserting oxygen O1 at the Wyckoff position 1*b* (½,½,½) with a resulting E_coh_./at.(B_12_O_2_:O) = −1.56 eV, a larger magnitude than all other hetero-inserted (X ≠ O) compounds but lower than pristine B_12_O_2_. Based on this observation, besides the fact that B_12_ is further stabilized through engaging iono-covalent bonds of one of the two B substructures with oxygen, namely B1–O, the insertion of additional central oxygen O1 is unfavorable versus B_12_O_2_ because it involves a competitive O1-O bonding versus B1-O on which the B_6_O structure is based, and hence weakening it. Indeed, the shortest X-O distance is observed for B_12_O_3_ with d(O1-O) = 1.73 Å (cf. Table 1), leading to suggest that O-O-O should be favored over O-X-O, independently of the chemical nature of X as observed from the respective cohesive energies.

Another argument can be evoked, pertaining to the fact that linear O_3_ is an unfavorable geometry both in the solid state and the isolated molecule. The angle O-O1-O amounts to 116.8°, smaller than the one identified by X-ray powder diffraction of solid ozone, amounting to 123.2° [27]. Both magnitudes are quite far from 180° characterizing a linear arrangement as in azide N_3_^−^ anion or in CO_2_, both with VEC = 16 electrons. Therefore, aligning three oxygen atoms in B_12_ host cavity (cf. Figure 1) is found less favorable energetically than the presence of only two oxygen atoms attached to one of the boron substructures, namely B1, arriving at B_12_O_2_ (or 2 B_6_O). Nevertheless, B_12_O_3_ is found largely cohesive. Such a favorable energy situation can be addressed through the bonding of the two end oxygens with one of the boron substructures B1 in view of their respective electronegativities: χ_O_ = 3.44 versus χ_B_ = 2.04.

The cohesive energies hierarchies are then chemically assessed in absolute magnitudes as: |E_coh_./at.(B_12_)| < |E_coh_./at.(B_12_O_3_)| < |E_coh_./at.(B_12_O_2_)|, on one hand and |E_coh_./at.(B_12_XO_2_) (X = B,C,N)| < |E_coh_./at.(B_12_O_3_)| on the other hand.

Lastly, the relatively E_coh_./at.(B_12_O_3_) large magnitude suggests the potential formation of this sub-oxide under optimized pressure/temperature conditions within the B_2_O_3_-B phase diagram [1]. Further emphasizing this relevant hypothesis, we calculated the cohesive energy of experimental B_12_C_3_ (B_4_C) also well known for use as an abrasive [13] and found a close magnitude of E_coh_./at.(B_12_C_3_) = −1.57 eV/at. This provides further support to our hypothesis of inserting X at the rhombohedron center on one hand and particularly oxygen with the potential existence of B_12_O_3_ on the other hand.

### 3.2. Trends of Valence Electron Count VEC

The valence electron count evoked above can help the further assessment of the electronic behavior in a preliminary step. For B, C, N, and O, VEC = 3, 4, 5, and 6, respectively. Then VEC(B_12_) = 36 (12 × 3). Focusing on the sub-oxides, VEC(B_12_O_2_) = 36 + 12 = 48 for B_12_O_2_ on one hand and VEC(B_12_O_3_) = 36 + 18 = 54 on the other hand, expecting closed shells. For illustration, the electronic density of states (DOS) projected over each constituent (PDOS) in Section 4 exhibits a small energy band gap of 1 eV between the filled valence band (VB) and the empty conduction band (CB) for both binary sub-oxides.

Regarding the interstitial B_12_O_2_:X, the atomic constituents: B(2s^2^, 2p^1^), C(2s^2^, 2p^2^), N(2s^2^, 2p^3^), and O(2s^2^,2p^4^) add on 3, 4, 5, and 6 valence electrons leading to VEC = 51, 52, 53, and 54, respectively, expecting a paramagnetic behavior for B and N as well as for the carbon case where the 2 p electrons remain unpaired.

### 3.3. Geometry Optimization Results

For B_12_O_2_, Table 1a presents the experimental and (calculated) structure results. A relatively good agreement is obtained for the lattice constants and the atomic positions for B1 and B2 substructures. This is also observed for the interatomic distances, the shortest one being B1-O at 1.49 Å.

Upon insertion of X at the body center the changes (Table 1b–e) are little for the B1 and B2 substructures but larger for *a_rh._* and oxygen coordinate *x*_O_ which increase regularly on the one hand and for the d(X-O) which decreases from 1.95 Å for X = B down to 1.81 Å for X = N, along with the slight decrease of the cohesive energy discussed above, on the other hand.

The exception for the largely cohesive B_12_O_3_ was discussed above in relation with its potential existence. NSP calculations were complemented with SP ones especially for X = B, C, and N. Only C and N showed a magnetically polarized ground state with ΔE(SP − NSP) = −0.53 and −0.34 eV, respectively, from the total energy calculations in the NSP and SP configurations and the subsequent development of a magnetic moment of 1.9 µ_B_ and 1 µ_B_—Bohr magneton—on C and N, respectively (vide infra). The reason why only one electron polarizes on nitrogen, is likely due to the bonding of the other two with oxygen of pristine B_12_O_2_ as illustrated by the strongest bonding between X and O observed relatively for N-O (cf. Section 4 illustrations) leaving a single non bonded electron and showing a significantly large density of states at the Fermi level, detailed in Section 4.

### 3.4. Electron Localization ELF Representations

Further illustration regarding the localization of the electrons and the corresponding bonding is obtained from the 2D (diagonal plane) and 3D ELF isosurfaces representing strong localization domains shown in grey volumes in Figure 2. As introduced in the Computation Section, ELF is a normalized function with 0 ≤ ELF ≤ 1. For the 2D ELF slice planes the ruler indicates the color code with 0 indicating zero localization with blue zones, ELF = ½ for free electron like behavior with green zones and ELF = 1 for full localization with red zones.

Figure 2a shows the ELF of B_12_O_2_ with a remarkable feature of a large blue zone between the two oxygen atoms surrounded by red ELF of strong localization. The two oxygens feature isolated grey volumes of non-bonded electrons pointing towards the central void, and they are expected to be involved with the X-O bonding. In Figure 2b, B in B_12_O_2_:B at the center fills the electron gap between the two oxygens while showing little localization around it. The introduction of the other X’s stresses the feature of larger localization and the occurrence of an increasingly large 3D grey torus around X (cf. Figure 2c–e) corresponding to non-bonding electrons. The emerging picture from the ELF representations is that besides showing the binding between O and B1 substructure, the presence of the X interstitial leads to the formation of a “3B1-O-X-O-3B1“-like linear complex along the rhombohedron diagonal, or along *c* if a hexagonal setup is considered (cf. Figure 1c).

### 3.5. Total and Magnetic Charge Densities

**Figure 3:**
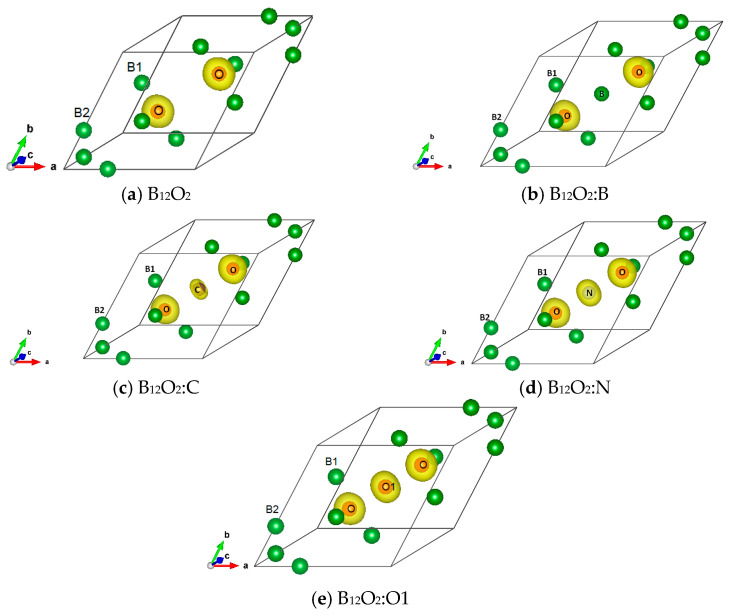
(color). Charge density projected onto the atomic constituents in (**a**) B_12_O_2_, (**b**) B_12_O_2_:B, (**c**) B_12_O_2_:C, (**d**) B_12_O_2_:N, and (**e**) B_12_O_2_:O1.

The charge density resulting from the self-consistent calculations for all B_12_O_2_:X are illustrated in Figure 3. The charge density projected onto the atomic constituents shows its prevalence on oxygen in the neighborhood of B1 substructure with no charge density on electron deficient boron, also observed in the central boron within B_12_O_2_:B. From X = C to O, there appears a charge density on X with an increasing size along with the increase of the number of valence electron, i.e., from four up to six.

**Figure 4:**
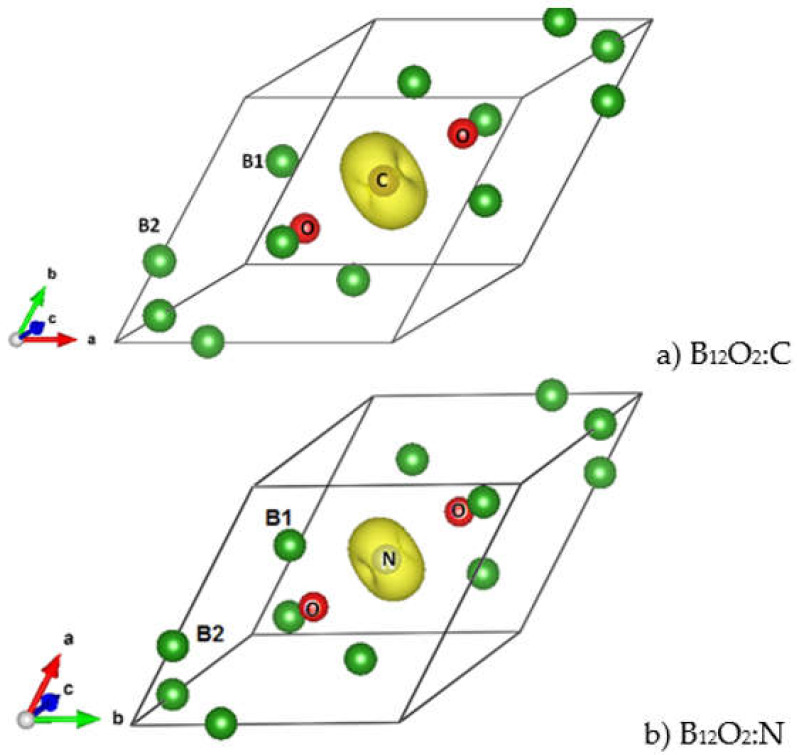
(color). Magnetic charge density in the two magnetically stable ternary compounds B_12_O_2_:C (**a**) and B_12_O_2_:N (**b**) exhibited by a torus centered on C and N and corresponding respectively to 1.9 μ_B_ and 1 μ_B_ magnetic moments.

Considering the VEC numbers of the different compositions and starting from the non-spin polarized NSP calculations, SP calculations were carried out by accounting for two spin channels, i.e., spin up ↑ and spin down ↓. The magnetic configuration was favored in the case of C and N providing a magnetic ground state and a magnetization M(B_12_O_2_C) = 1.9 µ_B_ and M(B_12_O_2_N) = 1 µ_B_. The atom projected magnetic charge density is shown in Figure 4 in the form of a torus identified only on the central interstitial atom, i.e., C and N with a larger volume on C proportionally to the twice-larger moment magnitude.

## 4. Electronic Structure and Bonding

The specific role of each chemical constituent in B_12_O_2_:X can be assigned based on the projection of the electronic density of states DOS and the COOP in the two magnetic configurations, NSP and SP, respectively. Using the data in Table 1, the calculations were carried out within the full potential augmented spherical wave (ASW) method [25,26] using the GGA gradient functional for the DFT XC effects [17]. The plots (in color) are shown with highlighting of the interstitial atoms partial DOS (PDOS) in red, then, the host ones subsequently.

### 4.1. NSP Calculations

**Figure 5:**
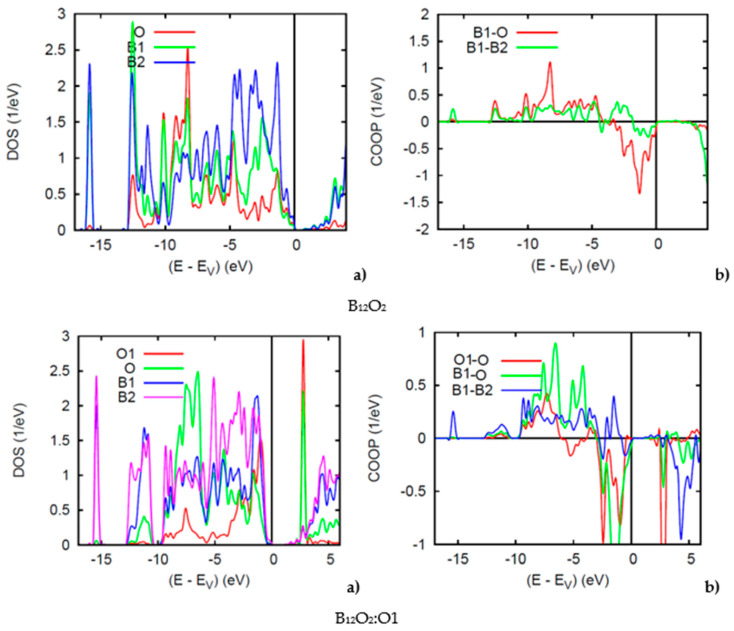
(color). (**a**) Site projected DOS and (**b**) chemical bonding from the COOP criterion of overlap valence populations in B_12_O_2_ (top) and B_12_O_2_:O1 (bottom).

Reporting firstly on the two binary sub-oxides, B_12_O_2_ and B_12_O_3_, Figure 5 shows the PDOS. Along the *x*-axis the energy zero is with respect to the top of the valence band VB E_V_ because both compounds are insulating with a small band gap of 1 eV for both binary oxides, in accordance with the even VEC count discussed above. In B_12_O_2_ the O and B1 PDOS skylines show resemblance oppositely to B2 which is far from O, at −16 eV there can be seen the B1(s) PDOS with negligible contribution from O—the B1 and O s states being at much lower energy (in Figure 5 (bottom)). Similar features are observed for B2 in B_12_O_3_ as with the B2(s) DOS at −16 eV. Also, a larger intensity O-PDOS is observed likely due to the charge density from O1 interstitial. In Figure 5, the conduction band shows a sharp peak corresponding to the O1-O interaction.

The COOPs shown on the right hand side further highlight the interactions between the chemical species. In B_12_O_2_ iono-covalent B1-O interaction exhibits a larger intensity than B1-B2. However, at the top of the VB it shows anti-bonding intensity due to the B1 bonding with O, competitive of the B1 bonding with the other boron substructure, B2. The COOP panel of B_12_O_2_ shows further features pertaining to the larger B1-O bonding following the PDOS larger magnitude of O. B1-B2 bonding is also prevailing with positive COOP intensities throughout the VB, thus ensuring the stability of the boron host structure B_12_. The negative COOP’s near the top of the VB are due to the competitive bonding of O with O1 versus B2.

**Figure 6:**
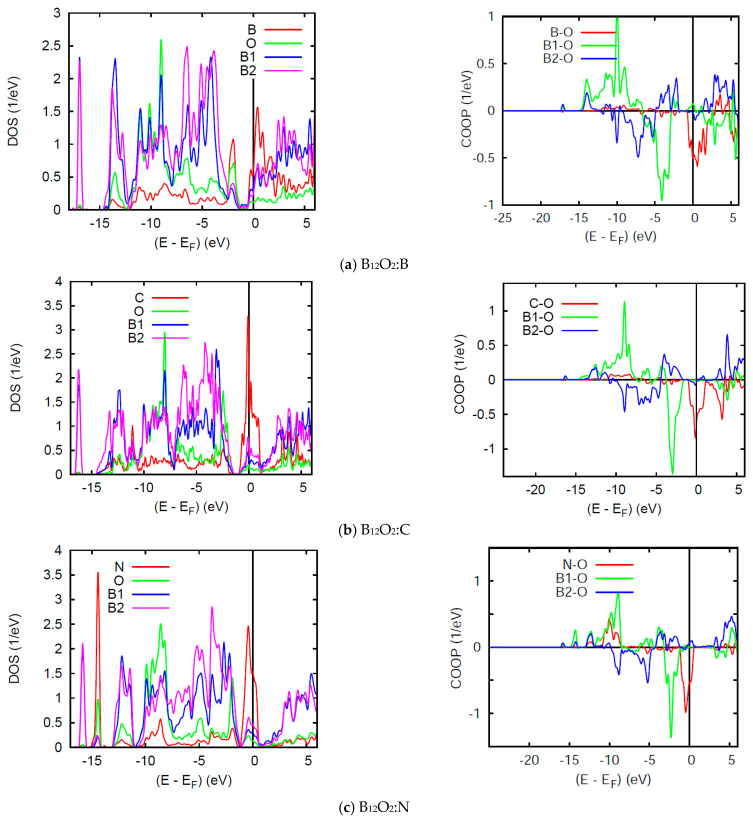
(color). B_12_O_2_:X. Site projected DOS (left) and chemical bonding from the COOP criterion (right) for (**a**) X = B; (**b**) X = C, and (**c**) X = N.

The DOS and COOP panels in Figure 6 exhibit a loss of the band gap observed in the binary sub-oxides and the energy reference is now with respect to the Fermi level E_F_. While other PDOS features are similar to the above discussion, we focus on the DOS at E_F_: *n*(E_F_) in such spin degenerate NSP calculations. All three ternary compounds show a relatively large *n*(E_F_) due to the interstitial atoms, B, C, and N which bring 3, 4, and 5 extra valence electrons, but more specifically 1, 2, and 3 p electrons, respectively. In B_12_O_2_:B, B bringing one p electron shows a slight perturbation of the pristine B_12_O_2_ DOS of Figure 5a with a small *n*(E_F_) intensity from one p electron. More significant *n*(E_F_) intensities are observed for B_12_O_2_:C and B_12_O_2_:N (Figure 6). Such high PDOSs are significant of electronic system instability in such total spins configuration assessed in the framework of the Stoner theory of band ferromagnetism explained in the textbook of Peter Mohn [28] and detailed in a case study in ref. [29]. Then the NSP configuration is not the ground state one and further SP calculations are needed. The VASP calculations above have actually shown that the SP configuration was the ground state with the onset of finite moments on C and N.

The right hand side COOP panels in Figure 6 show an increasing X-O COOP intensity along the series together with prevailing B1-O bonding and the competitive anti-bonding COOP near the top of the VB. Note that the large *n*(E_F_) correspond to anti-bonding states and stress further the electronic instability of the system in NSP configuration, meaning that the electronic system should stabilize upon accounting for two spin channels, majority spins (↑) and minority spins (↓) in SP calculations. Lastly, while negligible B-O and C-O COOPs are observed around −10 eV within the VB, relatively large COOP intensity is observed for N-O with a peak following the B1-O COOP shape. This implies a larger number of p- electrons from N involved with the bonding, namely two of them leaving one electron to spin polarize as shown below.

### 4.2. SP Calculations

SP calculations of B_12_O_2_:B did not lead to the onset of magnetization, in agreement with the VASP calculations. Expectedly, from the large C and N *n*(E_F_), both B_12_O_2_:C and B_12_O_2_:N led to magnetic solutions with a stable magnetization of 1.9 and 1 µ_B_, respectively. From the FP-ASW calculations using Table 1 data, the SP-NSP energy differences are: ΔE(SP − NSP) = −0.62 eV for B_12_O_2_:C and −0.37 for B_12_O_2_:N. These magnitudes are expectedly different from those obtained with the different method VASP (Section 3) where ΔE(SP − NSP) = −0.53 and −0.34 eV, respectively, but they remain within range, thus confirming the trend towards SP ground state.

While the two p electrons of carbon polarize due to sufficient localization, nitrogen has only one electron carrying the magnetic moment as assessed in the paragraph above and in Section 3.3 and Section 4.1.

**Figure 7:**
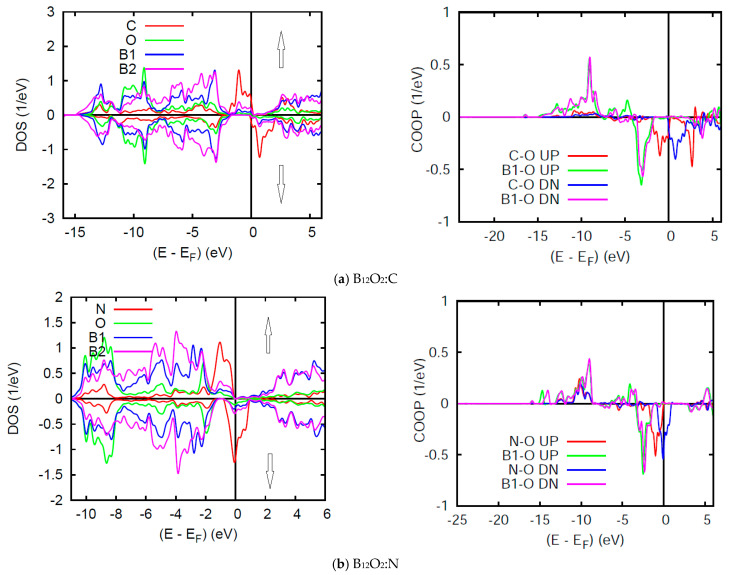
(color)**.** B_12_O_2_:X. Site and spin projected DOS and COOP in (**a**) X = C, and (**b**) X = N corresponding to 1.9 and 1 μ_B_, respectively.

Figure 7 shows the SP DOS in two subpanels for majority spins (↑) and minority spins (↓), named after their content of larger and smaller electron numbers as one may observe upon visual inspection of the energy downshift of the former (↑) and the energy up-shift for the latter (↓). However, such energy shift affects mainly the *n*(E_F_) states leading to a nearly insulating B_12_O_2_:C with very small *n*(E_F_) for both spin channels ↑, ↓ and non-negligible *n*_↓_(E_F_) in B_12_O_2_:N (half-filled band) which can be qualified as a half metallic ferromagnet. It needs to be highlighted here that such magnetic features are not unique in as far as they were also observed for transition metal ions as Cr in the rare transition metal oxide ferromagnet CrO_2_ which exhibits a half-metallic ferromagnetic ground state computed by us within DFT back in 1992 [30]. However, it was discussed earlier in a phenomenological schematic in 1973, by John B. Goodenough, in his text book on transition metal oxides [31].

The SP COOPs reflect the SP-DOS mainly through the energy shifts of the UP ↑ and DOWN (DN ↓) spin COOP shown on the right hand side panels of Figure 7. The main observation is the lowering of the anti-bonding COOP at E_F_.

Lastly, accounting for a possible anti-ferromagnetic state, complementary calculations were done through a doubling of the unit cell leading to two magnetic sub-cells with the first one considered as SPIN-UP and the second as SPIN-DOWN. The calculations led to an increase in the total energy and a decrease in magnetization, thus confirming the ferromagnetic ground state.

Nevertheless, it needs to be stressed that the calculations within DFT are at zero Kelvin implicitly. Since ΔG = ΔH − T.ΔS, then T.ΔS = 0 and the free energy ΔG corresponds to the enthalpy ΔH. Experimentally, the thermal effects are likely to play a significant role in the magnetic properties, such as the passage from ferromagnetic order to paramagnetic disorder state.

Syntheses efforts and subsequent measurements at low temperatures are likely to bring further assessment and clarification to the observations and results reported herein.

## 5. Conclusions

Based on experimental observations of *rh*.-B_12_O_2_, its expression as B_12_{O:X:O} lets one identify potential linear tri-atomic arrangement of atoms where X stands for a vacant position alike other compounds as B_12_C_3_ expressed as B_12_{C-C-C}, better known as B_4_C. With X interstitials belonging to the 1st period, *rh*.-B_12_O_2_:X (X = B, C, N, O) form a new family of ternary boron sub-oxides that we investigated within quantum theoretical DFT based on two complementary methods, namely a plane-wave one allowing for geometry optimizations and a second one, the augmented spherical wave (ASW) for detailed atom projected DOS and bonding assessments. The resulting compounds were found cohesive, albeit with smaller magnitude than pristine B_12_O_2_. The largest cohesion magnitude within the family was found for B_12_{O-O-O} or B_12_O_3_ translating the possibility of its synthesis under P and T conditions, alike the well-known abrasive B_4_C. B_12_O_2_ and B_12_O_3_ were found semi-conducting with a small band gap of 1 eV. Oppositely, the other B_12_O_2_:X (X = B, C, and N) were found metallic-like from the finite density of state at the Fermi level: *n*(E_F_). Large *n*(E_F_) magnitudes were identified for C and N in non-spin-polarized NSP calculations. Upon allowing for spin polarization SP, the onset of magnetization was found with M(B_12_O_2_C) = 1.9 µ_B_ and M(B_12_O_2_N) = 1 µ_B_. Their ground state was identified energetically to be ferromagnetic versus anti-ferromagnetic configuration. The energy and crystal chemistry numerical observations were further illustrated with total and magnetic charge density projections, as well as with the electron localization function (ELF) mapping and overlap population based COOP. In view of the relevant electronic and potential magnetic properties, such compounds could be prepared. Especially with the E_coh_./at.(B_12_O_3_) large magnitude (1.56 eV) relatively to the other B_12_O_3_:X compositions but close to E_coh_./at.(B_4_C) = 1.57 eV, it can be suggested the potential synthesis of B_12_O_3_ under specific pressure/temperature conditions within the B_2_O_3_-B phase diagram. Such P,T experiments using Flash Spark Plasma SPS [32] sintering are underway at the ICMCB-CNRS-University of Bordeaux.

## Figures and Tables

**Table:** (**a**) B_6_O or B_12_O_2_. Experimental [2] and (calculated) crystal parameters. *a_rh_* = 5.15 (5.13) Å; α = 63.04° (63.19)°.

Atom	Wyckoff	x	y	z
B_1_	6h	0.800 (0.804)	0.323 (0.323)	x
B_2_	6h	0.347 (0.333)	0.002 (0.003)	y
O	2c	0.376 (0.377)	x	x

d(O-O) = 3.06 (3.046); d(B1-O) = 1.49 (1.489); d(B1-B2) = 1.78 (1.82).

**Table:** (**b**) B_12_O_2_:B. Calculated crystal parameters; B at (1*b*) ½, ½, ½. *a_rh_* = 5.32 Å; α = 63.04°.

Atom	Wyckoff	x	y	z
B_1_	6h	0.806	0.321	x
B_2_	6h	0.333	0.003	y
O	2c	0.348	x	x

d(O-O) = 3.06 (3.046); d(B1-O) = 1.49 (1.489); d(B1-B2) = 1.78 (1.82).

**Table:** (**c**) B_12_O_2_:C calculated crystal parameters (non-spin polarized (NSP)/SP close values); C at (1*b*) ½, ½, ½. *a_rh_* = 5.30 Å; α = 61.97°.

Atom	Wyckoff	x	y	z
B_1_	6h	0.805	0.323	x
B_2_	6h	0.333	0.004	y
O	2c	0.352	x	x

d(C-O) = 1.88; d(B1-O) = 1.52; d(B1-B2) = 1.83.

**Table:** (**d**) B_12_O_2_:N calculated crystal parameters (NSP/SP close values); N at (1*b*) ½, ½, ½. *a_rh_* = 5.26 Å; α = 62.42°.

Atom	Wyckoff	x	y	z
B_1_	6h	0.804	0.321	x
B_2_	6h	0.333	0.003	y
O	2c	0.357	x	x

d(N-O) = 1.81, d(B1-O) = 1.52; d(B1-B2) = 1.73.

**Table:** (**e**) B_12_O_2_:O (Also expressed as B_12_O_3_). Calculated crystal parameters O1 at (1*b*) ½, ½, ½; *a_rh_* = 5.25 Å; α = 62.55°.

Atom	Wyckoff	x	y	z
B_1_	6h	0.804	0.320	x
B_2_	6h	0.332	0.003	y
O	2c	0.363	x	x

d(O1-O) = 1.73; d(B1-O) = 1.525; d(B1-B2) = 1.81.

## Data Availability

Data can be made available upon written request to the corresponding author and with a proper justification.
